# Promoting Appropriate Use of Drugs in Children

**DOI:** 10.1155/2012/906570

**Published:** 2012-05-08

**Authors:** Vijay N. Yewale, Dhanya Dharmapalan

**Affiliations:** General Pediatric Division, Dr. Yewale's Multispeciality Hospital for Children, Vashi, Navi Mumbai 400703, India

## Abstract

Promotion of appropriate and safe drugs in children is the need of the hour globally. Pediatric population by itself is a spectrum of different physiologies with significant variation in pharmacodynamics and pharmacokinetics. Unfortunately, 50–90% of drugs used in children today have never been actually studied in this population, and the results of drug studies done in adults are often extrapolated for use in children. Many medicines in pediatrics are off label or unlicensed. There is a spurt in drug resistance due to the overzealous prescription of antimicrobials not indicated, such as, using inadequate dosage or duration of drug regime leading to partially treated infections, using the wrong antimicrobial due to ignorance of causative organism, and finally using indigenous, irrational combinations. Availability of properly labeled and safe pediatric formulations, regular audit by pharmacists, judicious prescriptions, proper counseling about drug administration, surveillance of adverse effects, and pediatric drug trials can be the best possible interventions to offer appropriate medicines to children and thereby save millions of lives.

## 1. Introduction

Globally nearly nine million children under five years of age die every year, with pneumonia, diarrhea, and neonatal causes being the major killers [1]. Many of these conditions could be treated with safe, effective medicines. On the other hand, irrational use of the available drugs has led to adverse drug reactions and drug resistance to the usual pathogens and infections by unusual organisms. Promotion of appropriate and safe drugs in children is the need of the hour globally. 

## 2. Child: Not a Miniature Adult

Significant changes in the pharmacokinetics and pharmacodynamics occur as preterm infants mature toward term, as infants mature during the first few years of life, and as children reach puberty and adolescence. Hence pediatric population by itself is a spectrum of different physiologies and caters to different subgroups by age, namely, preterm neonates, full term neonates, infants and toddlers, and older children and adolescents.

The most notable differences in drug disposition between infants and children when compared with neonates and young adults centre around alterations in body water and serum protein composition [2].

Thereby a drug, unlike in an adult, is so very individual child specific, specific to the growth and development. Ignorance of this fact has caused adverse effects, for example, gray-baby syndrome caused by chloramphenicol.

Unfortunately, nowadays, 50–90% of drugs used in children have never been actually studied in this population. Prelicensure studies on safety and efficacy of a drug are generally carried out in adult subjects. In majority of times their safety and tolerability are extrapolated from such adult studies. The allometric methods used to calculate the dose for a child taking into account different categories of age, the body weight, and/or the body surface area consider the children as small adults, which is not the case [3].

## 3. The Ideal Children's Medicine

WHO states that “The ideal children's medicine is one that suits the age, physiological condition, and body weight of the child taking them and is available in a flexible solid oral dosage form that can be taken whole, dissolved in a variety of liquids, or sprinkled on foods, making it easier for children to take [4]”.

## 4. Rational Use of Drugs

Rational use of drugs can be defined as prescribing the right drug, in adequate dose for the sufficient duration and appropriate to the clinical needs of the patients at lowest cost [5]. 

The following measures can help in promoting appropriate and rational drugs in children.

### 4.1. Prescribing the Appropriate Medicines

All drugs have a therapeutic range below which they do not work or above which they are toxic. Drugs hence need to be used in the utmost rational manner in the correct dosages for correct duration. Though pediatrics is a well-recognized specialty, majority of the children in the health care system are being treated by family physicians who may not have received a good formal training in this field. This has led to wrong diagnosis and misuse of antibiotics and other drugs.

#### 4.1.1. Daily Practice Medicines

Most of the respiratory and gastrointestinal infections seen in day-to-day clinical practice are fortunately viral in origin and need only symptomatic treatment. Paracetamol is the safest antipyretic. The Indian medical fraternity has woken up to the rampant use of unsafe antipyretics and cough cold medicines and was taken steps to curb their usage. The latest examples are the ban of drugs like nimesulide and phenylpropanolamine containing anticold formulation, and so forth. The incidence of Reye's syndrome has significantly reduced, parallel to reduced use of aspirin. Many Western countries like the US and the UK have banned the use of cough-cold medicines for age below two years because of reports of lethal sedation due to accidental overdosage in this age group.

#### 4.1.2. Antimicrobials

Antimicrobials need a special mention due to the biggest threat of growing resistance to these drugs. The main reason behind this potential medical catastrophe is overzealous prescription of antimicrobials that are not indicated, for example in many viral infections, using inadequate dosage or duration of drug regime leading to partially treated infections, using the wrong antimicrobial due to ignorance of causative organisms, and finally using indigenous, irrational combinations.

Antibiotics should be prescribed as per the most probable causative agent causing the diseases. It is preferable to choose a single drug with the narrowest spectrum effective for the pathogen. Such a choice is likely to have the least toxicity, least cost, the least danger of inducing drug resistance to broad spectrum antibiotics, and the least chance of causing superadded infection with resistant organisms. One also needs to consider the effect of drug on the target site, for example the CSF permeability in case of meningitis.

While prescribing antibiotics, the host factors also need to be considered. For example, tetracycline should be given only above 7 years of age. Drug dosages need to be adjusted in hepatic or renal dysfunction. A bactericidal drug is preferred in an immunocompromised child.

Combination antibiotic therapy may be used if it provides synergy as in case of penicillin combination with an aminoglycoside for treatment of *S. viridans* endocarditis; or in polymicrobial infections like cerebral abscess, intraabdominal infections, and in prolonged courses given over months, for example, tuberculosis, leprosy, HIV infection to prevent emergence of drug resistant strains.

In situations where there is suspicion of infection based on indirect evidences of infection like leucocytosis (or leucopenia in neonates), acute phase reactants (like CRP, Calcitonin, etc.), radiology (consolidation on X-ray), and exudates (pleural fluid, CSF, joint aspirates, abscesses, etc.), antibiotics can be used empirically without awaiting definite identification of the causative organism after sending investigations aimed at making a microbiological diagnosis (if available and feasible). It is particularly important to send cultures diligently collected, before the first dose of any antibiotic is administered except in sick neonates or toxic child where one cannot wait for culture reports and need to start antibiotic urgently.

Patients requiring hospitalization for complicated or serious community acquired infections such as typhoid fever, pneumonia, or dysentery should be treated with first line drugs based on local epidemiology and prevailing sensitivity trends. It is best to use a single antibiotic. One may ideally choose an antibiotic with both parenteral and oral formulations so that the same antibiotic may be continued orally after initial parenteral therapy, at the same time keeping in mind that such a switch over, that is, sequential parenteral to oral, is not recommended in some infections like acute bacterial meningitis.

Children with chronic conditions, such as HIV/AIDS, may have to take several medicines daily. For them, fixed-dose combination products with several medicines in one pill are best. However, very few fixed-dose combinations for children exist.

Unfortunately several indigenous combinations have flooded the pharmaceutical market. The concern of these combinations is that they have been launched without adequate research into pharmacokinetic compatibility of the partner drugs and clinical efficacy. Examples of such drugs are norfloxacin, metronidazole, ceftriaxone, and tazobactam.

However intelligent man tries to become by inventing the best possible diagnostic tools and drugs to combat the microbes, it's a head-to-head race where often the microbes manage to develop some sort of mechanism of resistance. And most of the time our loss to these microbes can be attributed to the injudicious, irrational, and overuse of antibiotics. The best examples are the recent menace of extended-spectrum betalactamases (ESBLs) and methicillin resistant staphylococcus aureus (MRSA), and metallobetalactamases. overuse of 3rd generation cephalosporins has been implicated in increasing proliferation of ESBL pathogens. Increasing number of multidrug resistant typhoid, tuberculosis, and malaria cases pose a nightmare for treating physicians.

### 4.2. Designing Better Pediatric Formulations

Different formulations are required for each subgroup which would address the drug metabolism, compliance, timing of drug administration, and reactions to drug use.

Also, children are at a higher risk to develop adverse reactions due to greater prevalence of multidrug therapy as in the neonatal intensive care unit, or in children with greater length of hospital stay as in children with congenital or chronic diseases.

Due to nonavailability of appropriate pediatric formulations, for example, certain antitubercular drugs, antiepileptic and the health care providers have to resort to administering crushed tablets or dissolving in solvents, which can lead to administration errors and sometimes interfere with bioavailability. Lessons need to be learnt from the tragic incident of 4 children under 36 months who died from choking on albendazole tablets during a deworming campaign in Ethiopia in 2007 [6]. WHO in December 2007 launched a campaign called “Make medicines child size” which raised awareness and stimulated action to improve the availability of safe, effective, and quality medicines for children.

### 4.3. Minimizing Drug Administration Errors

Due to the need to calculate drug dosage on every pediatric patient based on either the weight, body surface area, age, or their body condition, there is high risk of medication errors, especially with incorrect recordings of patients, weights and the arithmetical calculations involved. Dosing errors of 10-fold or greater can occur due to miscalculation of misplacement of a decimal point. For example, at a crucial moment of cardiac resuscitation adrenaline has to be administered 0.01 mL/kg of 1 : 1000 dilution intramuscularly or 0.1 mL/kg of 1 : 10000 dilutions through intravenous route and a vise-versa miscalculation can prove fatal.

Inspite of better devices like syringe pumps for accuracy of dosage, drug errors may occur due to poor nursing compliance with the pump specifications leading to either poor response or an unexpected adverse reaction. It is not uncommon misunderstand the prescription that parents of drops versus syrup where sometimes, for example, as in case paracetamol, the drops may contain five-times higher concentration per mL compared to syrup, and wrong interpretation can easily lead to drug overdose. In a review in UK, over an eight-year period (1993 to 2000), there were 81 medication-error incidents involving at least 1144 children. There were at least 29 deaths, nine of which involved neonates. The most frequent type of medication error involved an incorrect dose [7].

Therefore, the goal of a zero drug error rate should be aggressively sought, with systems in place that aim to eliminate the effects of inevitable human error. This involves review of the entire system from drug manufacture to drug administration, not by finding fault in the individual, but by identifying faults in the system and building into those system mechanisms for picking up faults before they occur [8].

### 4.4. Minimizing Adverse Effects

Children are often exposed to the risk of adverse drug events. Because children, especially the younger ones, are less articulate in describing symptoms, and their nonverbal communication is often misunderstood or ignored, even serious adverse reactions in children often go unreported to health practitioners or authorities. All countries should establish national and regional monitoring systems for the detection of serious adverse medicine reactions in children. When such reporting systems exist, it is crucial that manufacturers follow up on adverse reactions to their products once they are on the market.

### 4.5. Improving Drug Compliance

Taste, smell, color, consistency, dosing frequency, and cost affect patient compliance with the drug regimen. Spitting of foul-tasting medications is very common among infants and younger children. Compliance should be improved with correct prescription of the drug as well as correct counseling of caretakers administering the drug. The importance of proper counselling cannot be overstated. However, inspite of prescribing the medicine in the correct dose for the correct duration, the crying infant may just spit out some amount of medicine while defiantly accepting it. Therefore, in all practical sense, what fraction of the prescribed medicine is really swallowed by the child remains a mystery.

### 4.6. Making Drugs Available

Surveys conducted in over 40 low-income countries show that 44% of public sector and 65% of private sector outlets had the listed generic medicines in stock. Lack of medicines in the public sector forces patients to go without or purchase medicines from private sector outlets where generic medicines cost on average 610% more than their international reference price [9]. It is of paramount importance that at least the essential pediatric medicines are available in the public sector which caters to majority of the population.

### 4.7. Educating Parents and Caretakers

Self-medication by parents on advice of relatives or acquaintances, traditional medicines, or any over-the-counter medicine unsupervised by a health professional is rampant in the Indian community, for example, medicines to promote teething in infants. Some medicines require reconstitution before oral use. Sometimes these medicines are wrongly preserved and used beyond their expiry which is harmful.

### 4.8. Supervising Public Health Programs Involving Children

In public health programs, comorbidity or malnutrition can exacerbate toxicity. For example, dehydration is frequently associated with ibuprofen-induced renal failure and malnutrition with paracetamol hepatotoxicity. Hence vigilance by the health authorities is required to check the clinical eligibility and health priority of the child receiving the medication. Also these programs should involve proper training of health workers for correct administration of the drugs to children.

### 4.9. Legalising Drug Related Issues by Health Authorities

#### 4.9.1. Drug Regulatory Body in India

The Central Drug Standards and Control Organization (CDSCO) is the principal regulatory body, which functions under the Ministry of Health and Family Welfare, government of India. It ensures the approval, production, and marketing of quality drugs by the following:

- laying down the standard of drugs,
- regulating market authorisation of new drugs and clinical research,
- approving licenses to manufacture certain categories of drugs as Central Licence Approving Authority, that is, large volume parenterals and vaccines and sera,
- carrying out investigation and prosecution in respect of contravention of legal provisions and regulating the standards of imported drugs,
- pre- and post-licensing inspection, recalling of substandard drugs, publication of Indian Pharmacopoeia,
- monitoring adverse drug reactions (ADR) and

screening periodically the drug formulations available in Indian market.

#### 4.9.2. Law Related to Spurious Drugs in India [10]

The Drugs and Cosmetics (Amendment) Act, 2008, passed by the Parliament on 5 December 2008 provides deterrent penalties for offences relating to manufacture of spurious or adulterated drugs which have serious implications on public health. The penalty for manufacture of spurious or adulterated drugs has been enhanced to an imprisonment for a term which shall not be less than 10 years but which may extend to imprisonment for life and shall also be liable to fine which shall not be less than ten lakh rupees or three-times value of the drug confiscated, whichever is more. In certain cases offences have been made cognizable and nonfbailable. It also provides a tool of compounding of offences for dealing with certain minor offences.

### 4.10. Curbing Lucrative Offers from the Pharmaceutical Industry

Incentive schemes for doctors and pharmacy staff from the pharmaceutical industry in the form of expensive gifts, free travel and leisure trips, or travel and accommodation arrangements prove a big hurdle in the prescription of rational drug with specific request for encouragement of their products. These practices need to be discouraged by the law as small personal gains can lead to long-term adverse effect on the community like increased resistance due to promotion of irrational drugs by such unethical doctors.

### 4.11. Educating the Doctors, Paramedical Staff, and Health Workers

Basic education in rational prescribing of drugs right from the undergraduate and postgraduate training days is very important for a clear foundation. Continued medical education (CME), conferences, circulars, newsletters, e-letters, and journals provide important source of information to the practitioners for education and promotion of the rational use of drugs.

## 5. Off-Label Drugs: Still a Gray Area

Off-label (unlabelled or unapproved) use of an approved product refers to the use of an approved product in a scenario that is not included or is disclaimed in the product information. Studies throughout Europe have shown that at least one third of children in hospitals and up to 90% of neonates in a neonatal intensive care unit receive such drug prescriptions. The medicines that are most frequently used off label include analgesics, antibiotics, and bronchodilators [11].

A study conducted to determine the extent and nature of off-label drugs in pediatric ward of a tertiary health centre in India found the off-label-drug use rate was 1.74 ± 1.56 per patient. The maximum rate of off-label drugs was in infants (2.33/patient). “Alteration in dosage” was by far the commonest reason for off-label use, followed by “age” and “indication.” Furosemide (i.v.), diazepam (i.v.), cefotaxime (i.v.), ethambutol (tab), and prednisolone (tab) were the five commonest off-label drugs used in the study population [12].

 Inspite of the rampant use of off-label drugs across the world, researchers have not been able to identify whether the use of off-label drugs in certain situations are ineffective, unsafe, or may cause substantial benefit to the patients [13]. The legal implications of using off-label drugs are still not clear.

## 6. WHO Initiatives

Promoting appropriate and safe drugs for children is a global concern. In December 2007, WHO published its first ever model list of essential medicine list for children with more than 200 medicines, including HIV/AIDS treatment, vaccines, anesthetics, hormones, vitamins, and minerals. This serves as a reference for countries to develop national essential medicines lists, according to their specific public health needs. The list is updated every two years and has been recognized as a powerful tool to promote health equity. The second edition was published in April 2010.

In India, the establishment of essential medicines lists for children in two states, Orissa and Chhattisgarh, is currently under way. The Indian Academy of Paediatrics is also reviewing a list for implementation at a national level. This will allow for better selection and procurement of child medicines based on specific needs.

## 7. More Research Needed

Differences in therapeutic approaches in children are wide, which implies the need for harmonization. A formulary is required which, in addition to providing the therapeutic function and dosage of the drug, can also be a source of up-to-date and evidence-based information for the most common clinical problems in and out of hospital. Since most of the drugs have not been studied in this population, it is important to have proper research trials. Unfortunately children, especially in the underdeveloped countries, are often victims of unethical clinical trials. Therefore, pediatric research trials need to be conducted under governance of strict laws and regulations.

## 8. Conclusion

There are enough clinical and scientific grounds to understand the significant differences in the pharmacological science between adults and children and not merely extrapolate the results from adult studies. It is of paramount importance to strengthen the health system so that the individual child's medical need is both scientifically and ethically addressed right from the drug manufacture to its administration. Availability of properly labeled pediatric formulations, regular audit by pharmacists, judicious prescriptions, proper counseling about drug administration, and surveillance of adverse effects can save millions of children.
